# Breast adipocyte size associates with ipsilateral invasive breast cancer risk after ductal carcinoma in situ

**DOI:** 10.1038/s41523-021-00232-w

**Published:** 2021-03-22

**Authors:** Mathilde M. M. Almekinders, Michael Schaapveld, Bram Thijssen, Lindy L. Visser, Tycho Bismeijer, Joyce Sanders, Edoardo Isnaldi, Ingrid Hofland, Marjolijn Mertz, Lodewyk F. A. Wessels, Annegien Broeks, Erik Hooijberg, Wilbert Zwart, Esther H. Lips, Grand Challenge PRECISION Consortium, Christine Desmedt, Jelle Wesseling

**Affiliations:** 1 Division of Molecular Pathology, Netherlands Cancer Institute, Amsterdam, The Netherlands; 2 Department of Pathology, Antoni van Leeuwenhoek Hospital, Amsterdam, The Netherlands; 3 Division of Psychosocial Research, Epidemiology and Biostatistics, Netherlands Cancer Institute, Amsterdam, The Netherlands; 4 Division of Molecular Carcinogenesis, Netherlands Cancer Institute, Amsterdam, The Netherlands; 5 Laboratory for Translational Breast Cancer Research, Department of Oncology, KU Leuven, Leuven, Belgium; 6 Department of Internal Medicine and Medical Specialties, Università degli Studi di Genova, IT-16132 Genova, Italy; 7 Core Facility Molecular Pathology and Biobanking, Netherlands Cancer Institute, Amsterdam, The Netherlands; 8 Bio-Imaging Facility, Netherlands Cancer Institute, Amsterdam, The Netherlands; 9 Oncode Institute, Utrecht, The Netherlands; 10 Division of Oncogenomics, Netherlands Cancer Institute, Amsterdam, The Netherlands; 11 Department of Pathology, Leiden University Medical Center, Leiden, The Netherlands

**Keywords:** Prognostic markers, Breast cancer, Risk factors, Cancer microenvironment, Tumour biomarkers

## Abstract

Although ductal carcinoma in situ (DCIS) is a non-obligate precursor to ipsilateral invasive breast cancer (iIBC), most DCIS lesions remain indolent. Hence, overdiagnosis and overtreatment of DCIS is a major concern. There is an urgent need for prognostic markers that can distinguish harmless from potentially hazardous DCIS. We hypothesised that features of the breast adipose tissue may be associated with risk of subsequent iIBC. We performed a case–control study nested in a population-based DCIS cohort, consisting of 2658 women diagnosed with primary DCIS between 1989 and 2005, uniformly treated with breast conserving surgery (BCS) alone. We assessed breast adipose features with digital pathology (HALO^®^, Indica Labs) and related these to iIBC risk in 108 women that developed subsequent iIBC (cases) and 168 women who did not (controls) by conditional logistic regression, accounting for clinicopathological and immunohistochemistry variables. Large breast adipocyte size was significantly associated with iIBC risk (odds ratio (OR) 2.75, 95% confidence interval (95% CI) = 1.25–6.05). High cyclooxygenase (COX)-2 protein expression in the DCIS cells was also associated with subsequent iIBC (OR 3.70 (95% CI = 1.59–8.64). DCIS with both high COX-2 expression and large breast adipocytes was associated with a 12-fold higher risk (OR 12.0, 95% CI = 3.10–46.3, *P* < 0.001) for subsequent iIBC compared with women with smaller adipocyte size and low COX-2 expression. Large breast adipocytes combined with high COX-2 expression in DCIS is associated with a high risk of subsequent iIBC. Besides COX-2, adipocyte size has the potential to improve clinical management in patients diagnosed with primary DCIS.

## Introduction

Ductal carcinoma in situ (DCIS) is a non-obligate precursor to invasive breast cancer (IBC). DCIS incidence has increased manifold since the implementation of population-based mammographic breast cancer screening^1–4^. In Western countries, DCIS comprises 15–30% of all newly diagnosed breast neoplasms^3–6^. DCIS is generally treated with surgery, often followed by radiotherapy and sometimes adjuvant hormonal therapy. The majority of patients with primary DCIS, however, will not develop a subsequent ipsilateral invasive breast cancer (iIBC)^7–10^, suggesting overdiagnosis and overtreatment. Clinicians urgently need prognostic biomarkers to distinguish hazardous from indolent DCIS for adequate risk stratification. Previous studies identified several clinicopathological prognostic factors for subsequent in situ or IBC recurrences^11–24^. A previous study in our Dutch nationwide DCIS cohort revealed human epidermal growth factor receptor 2 (HER2) overexpression, high cyclooxygenase (COX)-2 protein expression and the presence of periductal fibrosis as promising markers for predicting subsequent iIBC after primary DCIS^13^.

Obesity prevalence is rapidly increasing^25^ and is associated with an increased risk for developing postmenopausal IBC^26–31^ and poorer outcome in pre- and postmenopausal breast cancer^32,33^. Obesity at initial DCIS diagnosis has also been associated with increased risk of second breast cancers^34^.

Body mass index (BMI) alone is not sufficient for assessing adipose-tissue-related risk of IBC, as some postmenopausal women with higher body fat level have an elevated risk for IBC, despite of a normal BMI^35^. Increased adiposity is associated with (breast) adipocyte hypertrophy and low-grade inflammation of white adipose tissue (WAT), resulting from hypertrophic dying adipocytes encircled by macrophages forming crown-like structures (CLS)^29,36,37^. Although present in the majority of obese and overweight individuals^38^, enlarged adipocytes and WAT inflammation have also been found in the breast tissue of women with a normal BMI^39^. Apart from chronic inflammation of WAT, hyperadiposity commonly leads to altered local steroid hormone biosynthesis and disruptions in adipokine levels and insulin metabolism^40–43^. The altered microenvironment of (mammary) hyperadiposity has been associated with increased breast cancer risk^44^.

Although adipose tissue is a major component of the mammary gland, its potential role in development of subsequent iIBC after primary DCIS has received little attention. We hypothesised that breast adipocyte hypertrophy in DCIS is associated with a risk of subsequent iIBC.

## Results

### Patient and baseline characteristics

**Fig. 1:**
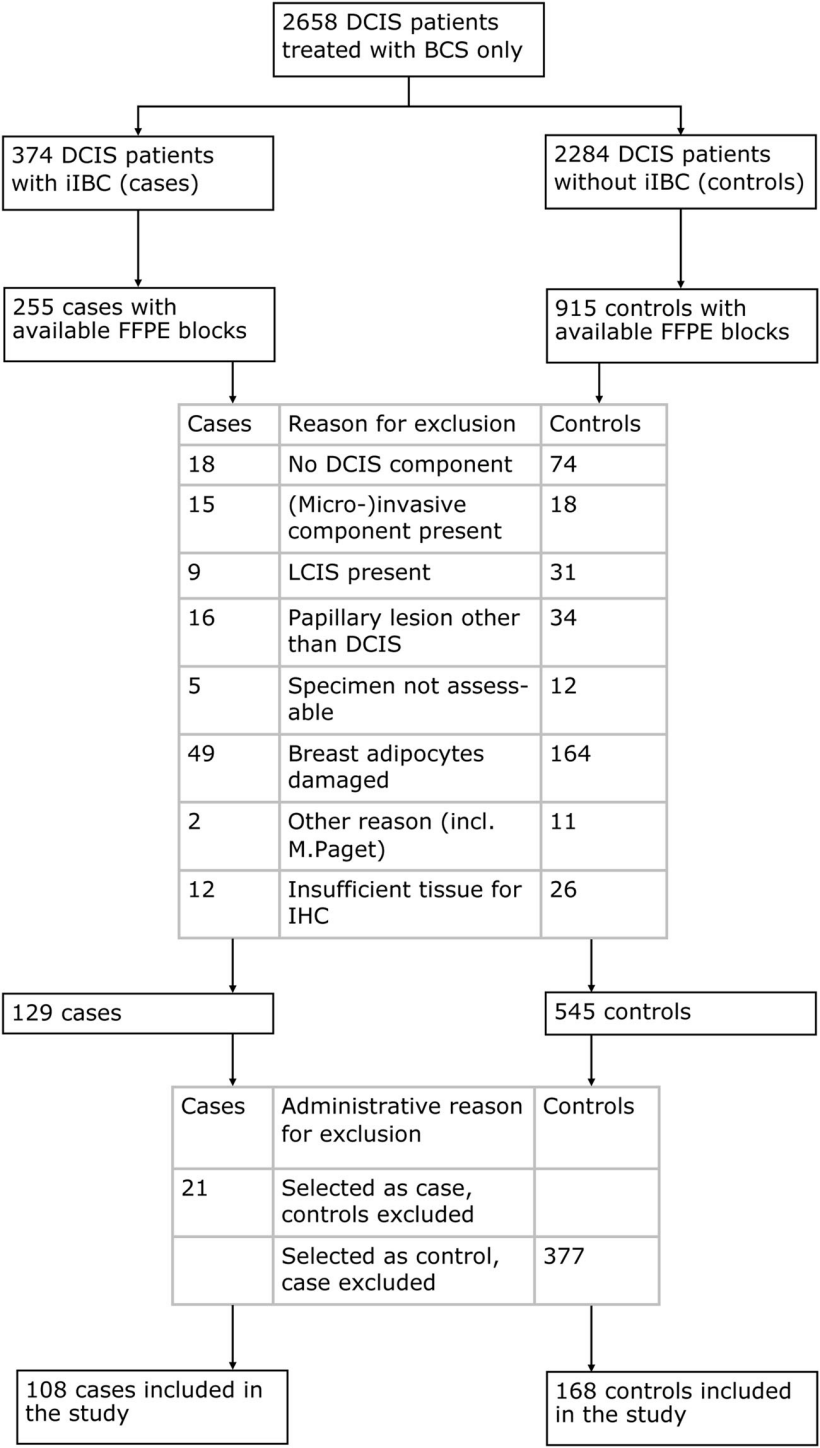
Diagram of women with DCIS included in the analysis. Abbreviations BCS: breast conserving surgery; iIBC: ipsilateral invasive breast cancer; FFPE: formalin-fixed paraffin-embedded; LCIS: lobular carcinoma in situ; IHC: immunohistochemistry. The cohort consisted of 2658 women with primary DCIS treated with BCS alone, of which 374 women developed subsequent iIBC, as first invasive cancer. At the start of the study, we received FFPE blocks of 255 and 915 controls. Some patients were excluded because no matched case or control was available for the case–control set they were part of. In total, 108 cases and 168 controls were included in the study.

Basic characteristics of our nested case–control study within a nationwide population-based cohort are described in the Methods section. A total of 276 DCIS patients that have undergone breast conserving surgery (BCS) alone were included in this case–control study (Fig. 1). Cases (*n* = 108) are women with specimen histology showing pure DCIS that predates diagnosis of iIBC. Controls (*n* = 168) are women with pure DCIS that have not developed subsequent iIBC and are matched for age of DCIS diagnosis (± 0 to 6 months). Median time from primary DCIS diagnosis to iIBC was 5.8 years (range 0.5–19.2). Clinical characteristics were comparable across cases and controls (Table 1). As the majority of DCIS patients was diagnosed during the implementation phase of mammographic screening (1989–1998), the proportion detected by mammographic screening was only 48%.

**Table 1:** Baseline clinical characteristics of women with primary DCIS.

Characteristics	DCIS cases (*n* = 108)	DCIS controls (*n* = 168)
	*n* (%)	*n* (%)
Age at diagnosis (years)		
<40	8 (7.4)	11 (6.5)
40–49	17 (15.7)	23 (13.7)
50–59	36 (33.3)	61 (36.3)
60–69	37 (34.3)	54 (32.1)
70–79	8 (7.4)	14 (8.3)
≥80	2 (1.9)	5 (3.0)
Year of DCIS diagnosis, mean (range)	1996 (1989–2004)	1997 (1989–2004)
Period of DCIS diagnosis^a^		
1989–1998 (screening implementation phase)	76 (70.4)	113 (67.3)
1999–2004 (full nationwide coverage)	32 (29.6)	55 (32.7)
Clinical presentation of DCIS		
Screen-detected	52 (48.1)	81 (48.2)
Non-screening-related	50 (46.3)	72 (42.9)
Unknown	6 (5.6)	15 (8.9)
Time to iIBC, mean in years (range)	5.8 (0.5–19.2)	–

*DCIS* ductal carcinoma in situ, *iIBC* ipsilateral invasive breast cancer. Women were treated with BCS (breast conserving surgery) alone, and subsequently developed (DCIS cases) or did not develop (DCIS controls) subsequent iIBC. Controls were matched to cases on age at diagnosis using a variable matching ratio. Controls were followed-up at least as long as the case they were matched to.

^a^Time of DCIS diagnosis was divided into two time periods based on the gradual implementation of the national breast cancer screening programme in the Netherlands for women >50 years of age: 1989–1998, corresponding to the implementation phase of the Dutch mammographic screening programme; and 1999–2004, when the screening programme was fully implemented.

Histologic grade was comparable between cases and controls. Grade 1 was found in 13.9% of cases and 13.1% of controls, grade 2 in 56.5% of cases and 61.9% of controls and grade 3 in 29.6% of cases versus 25.0% of controls (Table 2). DCIS phenotype in terms of dominant growth pattern, lesion size, presence of necrosis, calcifications, oestrogen receptor (ER), progesterone receptor (PR) and HER2 did not differ between cases and controls (Table 2). Periductal fibrosis (*P* = 0.10) and positive margin status (*P* = 0.10) were somewhat more common among cases compared to controls. Both margin status and lesion size were not always reliably reported in these older patient series and for 16% and 67%, respectively, these data were missing.

**Table 2:** Univariate results of histopathological characteristics and IHC markers associated with subsequent iIBC.

	DCIS cases (*n* = 108)	DCIS controls (*n* = 168)		
	*n* (%)	*n* (%)	OR (95% CI)^a^	*P*^b^
*Histopathology*				
Lesion size, millimetre				0.34
Mean (range)	13 (2–30)	11 (2–50)	1.04/mm (0.96–1.13)	
Lesion size				0.47
≤10 mm	15 (13.9)	44 (26.2)	1.00 (reference)	
>10 mm	19 (17.6)	23 (13.7)	1.49 (0.51–4.31)	
Unknown	74 (68.5)	101 (60.1)		
Margin status				0.10
Free	48 (44.4)	102 (60.7)	1.00 (reference)	
Not free	33 (30.6)	50 (29.8)	1.63 (0.91–2.92)	
Unknown	27 (25.0)	16 (9.5)		
Dominant growth pattern^c^				0.90
Solid	71 (65.7)	103 (61.3)	1.00 (reference)	
Cribriform	21 (19.4)	34 (20.2)	0.92 (0.49–1.74)	
(Micro)papillary	7 (6.5)	11 (6.5)	0.91 (0.33–2.47)	
Clinging	9 (8.3)	20 (11.9)	0.65 (0.27–1.60)	
Histologic grade^d^				0.79
Grade 1	15 (13.9)	22 (13.1)	1.00 (reference)	
Grade 2	61 (56.5)	104 (61.9)	0.89 (0.43–1.83)	
Grade 3	32 (29.6)	42 (25.0)	1.07 (0.50–2.30)	
Necrosis				0.44
Absent	23 (21.3)	42 (25.0)	1.00 (reference)	
Present	85 (78.7)	126 (75.0)	1.25 (0.71–2.22)	
DCIS-associated calcifications				0.91
Absent	28 (25.9)	43 (25.6)	1.00 (reference)	
Present	80 (74.1)	125 (74.4)	1.03 (0.57–1.89)	
Periductal fibrosis				0.10
Absent	68 (63.0)	125 (74.4)	1.00 (reference)	
Present	40 (37.0)	42 (25.0)	1.57 (0.92–2.67)	
N/A	0	1 (0.6)		
Periductal lymphocytes				0.15
Absent/sparse	70 (64.8)	125 (74.4)	1.00 (reference)	
Prominent	38 (35.2)	43 (25.6)	1.45 (0.87–2.41)	
*Immunohistochemistry*				
ER^e^				0.71
Negative	25 (23.1)	34 (20.2)	1.00 (reference)	
Positive	83 (76.9)	134 (79.8)	0.89 (0.47–1.68)	
PR^e^				0.83
Negative	45 (41.7)	66 (39.3)	1.00 (reference)	
Positive	62 (57.4)	100 (59.5)	0.95 (0.58–1.55)	
N/A	1 (0.9)	2 (1.2)		
Her2				0.17
Negative	69 (63.9)	123 (73.2)	1.00 (reference)	
Positive	37 (34.3)	43 (25.6)	1.46 (0.84–2.54)	
N/A	2 (1.9)	2 (1.2)		
Subtypes				0.27
HR+ Her2-	66 (62.3)	113 (67.3)	1.00 (reference)	
HR+ Her2+	17 (15.7)	19 (11.3)	1.58 (0.75–3.29)	
HR- Her2+	20 (18.5)	24 (14.3)	1.26 (0.61–2.60)	
HR- Her2-	3 (2.8)	10 (6.0)	0.36 (0.075–1.73)	
N/A	2 (1.9)	2 (1.2)		
Cox-2				<0.001
Low	10 (9.3)	36 (21.4)	1.00 (reference)	
High	96 (88.9)	128 (76.2)	3.70 (1.59–8.64)	
N/A	2 (1.9)	4 (2.4)		

*N/A* not assessable, *HR+* ER positive and/or PR positive, *HR-* ER negative and PR negative. N/As, unknown lesion size and margin status were not included in the analysis.

^a^DCIS cases and DCIS controls were compared by univariate conditional logistic regression.

^b^*P*-values are likelihood ratio based.

^c^Among histopathological features, the variable “dominant growth pattern” was defined as the growth pattern (solid, cribriform, (micro)papillary or clinging) that comprises the largest proportion of the DCIS lesion if more than one growth pattern is present.

^d^Histologic grade was based on nuclear grade.

^e^ER and PR were considered positive when ≥10% of the luminal epithelial cells showed nuclear staining of any intensity.

Immunohistochemical COX-2 expression was scored as “low” or “high” and the scoring system was described in detail in the Methods section. COX-2 expression was significantly more often highly expressed in cases compared to controls (odds ratio (OR) 3.70, 95% confidence interval (95% CI) = 1.59–8.64, *P* < 0.001; Table 2).

### Breast adipocyte measurements

**Fig. 2:**
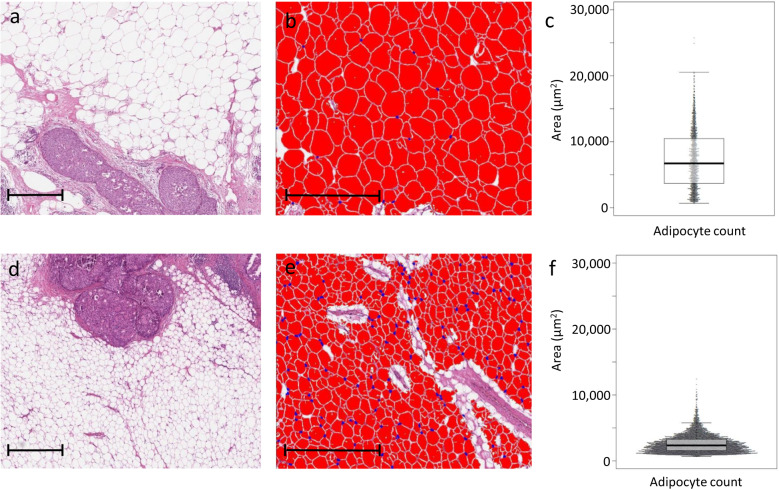
Digital assessment of breast adipocyte area in two representative patients. Scale bars represent 500 µm. **a** A 68-year-old patient with DCIS that developed subsequent iIBC. The breast adipose tissue consists of large adipocytes. **b** Adipocytes were analysed with the vacuole module v2.1 (HALO software, Indica Labs, Corrales, NM, USA). **c** Beeswarm plot of the corresponding distribution of adipocyte area with mean adipocyte area and area^75th^ of 7287 and 10493 µm^2^, respectively. **d** DCIS surrounded by small adipocytes from a 38-year-old patient. No subsequent iIBC occurred during follow-up. **e** Corresponding image of analysed adipocytes in HALO software. **f** Beeswarm plot of the corresponding distribution of adipocyte area with mean adipocyte area and area^75th^ of 2593 and 3274 µm^2^, respectively.

Breast WAT features including relative amount (percentage) of adipose tissue, adipocyte diameter at the 75^th^ percentile (adipocyte diameter^75th^) and adipocyte area at the 75^th^ percentile (adipocyte area^75th^) were assessed with digital pathology among all patients (see Methods). Median percentage of breast adipose tissue was 65% (interquartile range (IQR) 47–77%). A median number of 2514 adipocytes was measured per patient with digital pathology (IQR 1313–5419). Median adipocyte diameter at the 75^th^ percentile (adipocyte diameter^75th^) was 86 µm (IQR 77–94) and median adipocyte area at the 75^th^ percentile (adipocyte area^75th^) was 6174 µm^2^ (IQR 5165–7604), respectively. Figure 2 illustrates the appearance and distribution of adipocytes in two representative patients.

**Fig. 3:**
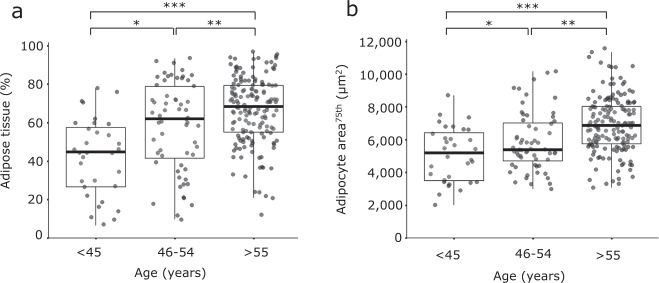
Older DCIS patients have a higher percentage of breast adipose tissue and larger adipocyte size. **a** A higher percentage of mammary adipose tissue in one representative slide per DCIS patient is significantly associated with older age in *n* = 249 (unique) DCIS patients. *P = 0.002, **P = 0.028, ***P < 0.001 (Mann-Whitney *U* Test), overall *P* < 0.001 (Kruskal-Wallis test). **b** Larger mammary adipocyte size is significantly associated with older age. *P = 0.049, **P < 0.001, ***P < 0.001 (Student’s *t*-test), overall *P* < 0.001 (one-way ANOVA).

Higher relative area of breast adipose tissue and larger adipocyte size were significantly associated with higher age (*P* < 0.001, Fig. 3 and Supplementary Fig. 1). The age group of >55 years, used as a proxy for postmenopausal status, shows a significantly higher relative area of mammary adipose tissue (*P* < 0.001) and larger adipocyte size (*P* < 0.001) than the age group of <45 years.

### Assessment of crown-like structures (CLS)

CLS assessment as a measure for WAT inflammation was possible for 56 DCIS patients (26 cases matched with 30 controls) for whom at least five paraffin blocks were available. Median number of CLS per 10 cm^2^ was 1.4 (range 0–211 CLS/10 cm^2^, IQR 0–8 CLS/10 cm^2^). The number of CLS per 10 cm^2^ correlated with adipocyte area^75th^ (ρ = 0.47, 95% CI = 0.23–0.66, *P* < 0.001, Supplementary Fig. 2). In the subset of DCIS patients for which CLS/10 cm^2^ was assessed, subsequent iIBC risk did not differ between patients with a high (≥5 CLS/10 cm^2^) and a low (<5 CLS/10 cm^2^) CLS count (OR 1.2, 95% CI = 0.43–3.35, Supplementary Table 2).

### Adipocyte characteristics associated with subsequent iIBC

Breast adipocyte size at primary DCIS diagnosis was positively associated with risk for a subsequent iIBC (Table 3). For each 10 µm increase in adipocyte diameter^75th^ (range 47–118 µm), the OR for a subsequent iIBC increased with 25% (95% CI = 1.02–1.54), while for each 10^3^ µm^2^ increase in adipocyte area^75th^ (range 2016–11569 µm^2^) the OR for subsequent iIBC increased with 17% (95% CI = 1.01–1.35). Women with an adipocyte area^75th^ within the highest quartile had a 2.75-fold (95% CI = 1.25–6.05) increased risk for subsequent iIBC compared to women with an adipocyte area^75th^ within the lowest quartile. When compared to women with an adipocyte area^75th^ within the lowest three quartiles, women with an adipocyte area^75th^ of the highest quartile had a 2.08 times (95% CI = 1.16–3.74) increased risk for subsequent iIBC. Relative area of breast adipose tissue was not associated with subsequent iIBC risk.

**Table 3:** Univariate and multivariate results of adipocyte characteristics and COX-2 expression in relation to subsequent iIBC.

Characteristics	Range	DCIS cases (*n* = 108)	DCIS controls (*n* = 168)		
		*n* (%)	*n* (%)	OR (95% CI)^a^	*P*^b^
*Univariate results*					
Adipocyte diameter^75th^	47–118 µm			1.25 (1.02–1.54) per 10 µm	0.030
Adipocyte area^75th^	2016–11,569 µm^2^			1.17 (1.01–1.35) per 10^3^ µm^2^	0.039
Adipocyte area^75th^					0.053
Quartile 1	2016–5168 µm^2^	24 (22.2)	45 (26.8)	1.00 (reference)	
Quartile 2	5169–6175 µm^2^	24 (22.2)	45 (26.8)	1.19 (0.56–2.53)	
Quartile 3	6176–7611 µm^2^	26 (24.1)	43 (25.6	1.65 (0.74–3.66)	
Quartile 4	7612–11,569 µm^2^	34 (31.5)	35 (20.8)	2.75 (1.25–6.05)	
Adipocyte area^75th^					0.013
Quartile 1–3	2016–7611 µm^2^	74 (68.5)	133 (79.2)	1.00 (reference)	
Quartile 4	7612–11,569 µm^2^	34 (31.5)	35 (20.8)	2.08 (1.16–3.74)	
Adipose tissue	6.6–96.5%			1.03 (0.91–1.17) per 10%	0.645
*Multivariate results*^c^					
Adipocyte area^75th^/COX-2					<0.001
Adipocyte area^75th^ COX-2	2016–11,569 µm^2^			1.20 (1.03–1.41) per 10^3^ µm^2^	
Low		10 (9.3)	36 (21.4)	1.00 (reference)	
High		96 (88.9)	128 (76.2)	4.06 (1.71–9.66)	
N/A^#^		2 (1.9)	4 (2.4)		
Adipocyte area^75th^/COX-2					<0.001
Adipocyte area^75th^					
Quartile 1	2016–5168 µm^2^	24 (22.2)	45 (26.8)	1.00 (reference)	
Quartile 2	5169–6175 µm^2^	24 (22.2)	45 (26.8)	1.13 (0.51–2.52)	
Quartile 3	6176–7611 µm^2^	26 (24.1)	43 (25.6	1.69 (0.74–3.88)	
Quartile 4	7612–11,569 µm^2^	34 (31.5)	35 (20.8)	3.05 (1.32–7.05)	
COX-2					
Low		10 (9.3)	36 (21.4)	1.00 (reference)	
High		96 (88.9)	128 (76.2)	4.33 (1.79–10.5)	
N/A^d^		2 (1.9)	4 (2.4)		
Adipocyte area^75th^/COX-2					<0.001
Adipocyte area^75th^					
Quartile 1–3	≤7611 µm^2^	74 (68.5)	133 (79.2)	1.00 (reference)	
Quartile 4	≥7612 µm^2^	34 (31.5)	35 (20.8)	2.34 (1.23–4.45)	
COX-2					
Low		10 (9.3)	36 (21.4)	1.00 (reference)	
High		96 (88.9)	128 (76.2)	4.24 (1.76–10.2)	
N/A^4^		2 (1.9)	4 (2.4)		
Adipocyte area^75th^ /COX-2					<0.001
Area_q1–3/low		5 (4.6)	26 (15.5)	1.00 (reference)	
Area_q1–3/high		68 (63.0)	104 (61.9)	5.56 (1.64–18.9)	
Area_q4/low		5 (4.6)	10 (6.0)	4.15 (0.75–22.9)	
Area_q4/high		28 (25.9)	24 (14.3)	12.0 (3.10–46.3)	
N/A^4^		2 (1.9)	4 (2.4)		

^a^DCIS cases and DCIS controls were compared by univariate or multivariate conditional logistic regression.

^b^*P*-values are likelihood ratio based.

^c^Adipocyte area^75th^ and COX-2 were included in multivariate analysis.

^d^Patients in which COX-2 immunohistochemistry was not assessable; N/As were not included in the analysis.

We also assessed whether adipocyte area^75th^ remained associated with iIBC risk when adjusted the presence of periductal fibrosis, Her2 status and COX-2 expression in DCIS cells (Table 2 and Supplementary Table 3). In the presence of high COX-2 expression, adipocyte area^75th^ remained an independent predictor of subsequent iIBC. Women with an adipocyte area^75th^ within the highest quartile had a 3.05-fold (95% CI = 1.32–7.05) increased risk for subsequent iIBC compared to women with an adipocyte area^75th^ within the lowest quartile, while COX-2 was an independent predictor for iIBC in the presence of adipocyte area^75th^ (OR 4.33, 95% CI 1.79–10.5). Compared to women with an adipocyte area^75th^ within quartiles 1–3, women with an adipocyte area^75th^ within the highest quartile had a 2.34-fold increased risk for subsequent iIBC (OR 2.34, 95% CI = 1.23–4.45, Table 3) while COX-2 was an independent predictor for iIBC in the presence of adipocyte area^75th^ (OR 4.24, 95% CI = 1.76–10.2). The risk of subsequent iIBC in DCIS patients with high COX-2 expression and an adipocyte area^75th^ within the highest quartile was 12 times higher than in DCIS patients with low COX-2 expression and an smaller (quartile 1–3) adipocyte area^75th^ (OR 12.0, 95% CI = 3.10–46.3, Table 3).

Periductal fibrosis and Her2 were no independent predictors in a model which already contained adipocyte area^75th^ and COX-2, and did not change the estimate for adipocyte area^75th^ and COX-2 (Supplementary Table 3).

**Fig. 4:**
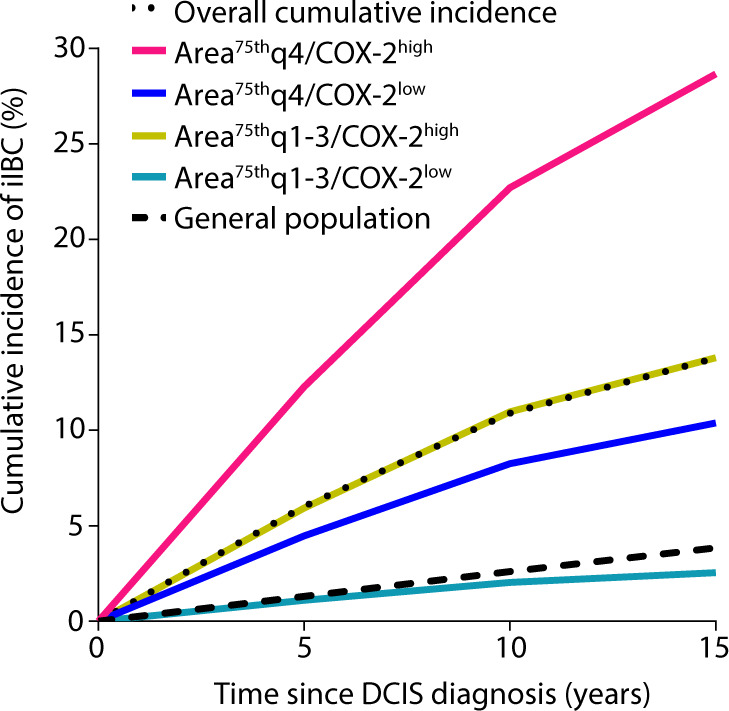
Estimated cumulative incidence of subsequent iIBC in DCIS for a combination of adipocyte area^75th^ and COX-2 status. Estimated cumulative incidence of iIBC per category of adipocyte area^75th^/COX-2 status among women with primary DCIS treated by BCS alone. Cumulative incidence of subsequent iIBC by adipocyte area^75th^ and COX-2 status was estimated using the ORs for adipocyte area^75th^ and COX-2 status from the current study. The cumulative risk of iIBC and death due to other causes was derived from the full cohort.

The estimated overall 10- and 15-year cumulative iIBC incidence in our study population was 10.9% and 13.8%, respectively (Fig. 4). DCIS patients with large breast adipocytes and high COX-2 expression (area^75th^q4/COX-2^high^) had a 10- and 15-year cumulative incidence of 22.7% and 28.7%, respectively (Fig. 4), while DCIS patients with smaller breast adipocytes (area^75th^q1–3) and low COX-2 expression had an estimated 10- and 15-year iIBC cumulative incidence of 2.0% and 2.6%, respectively. Within our study population, a combination of large adipocyte size and high COX-2 expression (area^75th^q4/COX-2^high^) was present in 25.9% of cases and 14.3% of controls (Table 3).

## Discussion

In this study we investigated mammary adiposity in relation to risk of subsequent iIBC after primary DCIS. We show that the presence of large breast adipocytes in primary DCIS is associated with a risk for subsequent iIBC. DCIS with high COX-2 expression, a cytoplasmic enzyme involved in prostaglandin synthesis, was also associated with iIBC risk, as we previously showed^13^. Moreover, in combination with high COX-2 expression in DCIS cells, patients with large breast adipocyte size (area^75th^, highest quartile) have a 12-fold increased risk for a subsequent iIBC, resulting in an estimated 10- and 15-year cumulative iIBC incidence of 22.7% and 28.7%, respectively. Women with smaller breast adipocytes (area^75th^, quartiles 1–3) in combination with low COX-2 have a 10- and 15-year cumulative iIBC incidence of 2.0% and 3.9%, respectively, which is similar to the 10- and 15-year breast cancer incidence in the general population. However, it should be noted that the DCIS patients in this lowest risk category have been treated (with BCS alone). Taken together, besides COX-2 expression, breast adipocyte size appears to be promising for clinical management in primary DCIS.

Several other important findings have emerged from this study. Firstly, we found that women with DCIS in the age group of <45 years on average have the smallest adipocytes and the lowest percentage of mammary adipose tissue among all age groups. DCIS patients in the age group >55 years (as a proxy of postmenopausal status) have the largest adipocytes and highest relative area of adipose tissue. The association between breast adipocyte characteristics and age underlines that matching DCIS cases and controls for age is essential. Iyengar et al. found a correlation between breast adipocyte size, CLS and postmenopausal status among breast cancer patients that underwent mastectomy^36^. As DCIS incidence sharply rises during menopause and is highest in the postmenopausal group (60% of all patients in our study are >55 years), future research may focus on the role of adipocytes in the pathogenesis of iIBC after primary DCIS in the context of the menopausal transition.

Secondly, we found that breast adipocyte size correlates with adipose inflammation expressed as the number of CLS per 10 cm^2^. Number of breast CLS and BMI previously has been shown to correlate with breast adipocyte diameter in IBC^36,39,45^. Carter et al. found that a high CLS count (>5 CLS/sample) was associated with subsequent IBC (OR 6.8, 95% CI = 1.4–32.4) in biopsies of 172 patients with benign breast disease^44^. In our study, number of CLS/10 cm^2^ was not associated with subsequent iIBC in 56 DCIS patients, although the analysis had low power to detect such an association.

WAT inflammation has been associated with elevated secretion of proinflammatory mediators and adipokines such as COX-2, TNF-α, IL-1ß and leptin^46,47^, resulting in the induction of aromatase, the rate-limiting enzyme for oestrogen biosynthesis^48^. The subsequent locally produced oestrogen may be a key driver to ER-positive IBC. In the present study, however, ER status alone was not a predictor of iIBC risk and did not significantly change the risk estimate when added to a model containing adipocyte area^75th^ and COX-2. The study potentially lacked power to show significant interaction. Larger studies and mechanistic studies are needed to investigate the relationship between WAT inflammation, COX-2, ER status and outcome after primary DCIS.

Our study presented some limitations. Firstly, BMI was not available from the old patient records from 1989–2004 in our study. However, a study of Iyengar et al. indicates that BMI is not always an appropriate proxy for breast cancer risk in postmenopausal women, because women with increased body fat as assessed by dual-energy X-ray absorptiometry (DXA) are at elevated risk of breast cancer despite of a normal BMI^35^. Furthermore, a second study of Iyengar et al. in women with a normal BMI undergoing mastectomy for breast cancer treatment or risk reduction showed WAT inflammation in 39% of women^39^. Since WAT inflammation was associated with larger breast adipocytes, higher circulating leptin levels and increased aromatase in breast tissue, breast adipocyte size may be more accurate for breast cancer risk assessment than BMI alone^39^.

Secondly, in 35% of the DCIS lesions, breast adipocytes were too severely damaged and therefore we were unable to assess adipocyte size in these patients. In addition, analyses of CLS were limited by the relatively low number of DCIS patients with ≥5 available formalin-fixed paraffin-embedded (FFPE) blocks. In order to increase the number of DCIS specimen with intact and sufficient breast adipose tissue, future studies on breast adipocytes could benefit from adaptations in the surgical breast pathology pipeline.

Thirdly, data on lesion size was often missing (67% of patients). Furthermore, patients with a positive margin status typically undergo a re-excision lumpectomy or mastectomy according to Dutch guidelines. The final margin status after re-excision was not reliably registered between 1989 and 2004. Among the patients with known lesion size and margin status, no significant differences were observed between cases and controls.

A major strength of our study is the population-based nested case–control design allowing for the study of a high number of subsequent iIBC with long-term follow-up. Treatment of DCIS with BCS alone was more commonly practiced between 1989 and 2004. All DCIS patients received a uniform treatment of BCS alone, without radiotherapy or endocrine therapy that could impact subsequent iIBC risk. Furthermore, the population-based nature and the fact that the patient-material was derived from 58 different pathology laboratories, makes our results potentially more generalisable.

The study is further strengthened by the use of digital pathology, enabling tissue segmentation and the automatic evaluation of the diameter and area of high numbers of intact adipocytes. The measurement of the adipocyte area rather than its diameter approximates the three-dimensional nature of an adipocyte in real life more accurately, as compared to manual measurements.

In conclusion, breast adipocyte area^75th^ and COX-2 are promising prognostic markers for prediction of iIBC risk in primary DCIS patients. Patients with an adipocyte area^75th^q4/COX-2^high^ DCIS lesions when treated with BCS alone, had a 12-fold increased risk for subsequent iIBC compared to adipocyte area^75th^q1-3/COX-2^low^ DCIS lesions, and thus may benefit from more careful monitoring during follow-up.

As a next step, our findings need to be confirmed in independent cohorts in which not only subsequent iIBC but also contralateral iIBC risk is considered^49^, after which our findings could be used to improve clinical management at primary DCIS diagnosis. Further studies are needed to unravel the exact underlying mechanisms behind the notable associations of mammary adiposity, adipocyte inflammation and anthropometric factors. The acquired insights may be used to reduce risk of subsequent iIBC after primary DCIS.

## Methods

### Study population

We conducted a nested case–control study within a nationwide population-based cohort of 2658 DCIS women diagnosed in the Netherlands with primary DCIS between 1 January 1989 and 31 December 2004, and a median follow-up of 12.0 years (IQR 9.0–15.3). Women were uniformly treated with BCS without radiotherapy and/or anti-hormonal therapy. A detailed description of this cohort was published previously^9,13^. Women with primary DCIS that developed iIBC were defined as cases. Controls correspond to women with primary DCIS that did not develop iIBC for at least the same follow-up duration as time to iIBC in the corresponding cases. Cases and controls were matched for age at DCIS diagnosis (±0 to 6 months). Cases and controls were not matched for year of diagnosis. None of the patients developed subsequent contralateral IBC while on study. FFPE tissue blocks and pathology reports were obtained from 58 pathology laboratories in the Netherlands^13^. Margin status and lesion size were not routinely registered in the pathology reports, especially not in the older cases of our retrospective series.

The study was approved by the review boards of the Netherlands Cancer Registry and the Dutch National Pathology Automated Archive (PALGA). The secondary use of tissue and data in this study is covered by an opt-out regimen conform Dutch regulations, the Code of Conduct of Federa-COREON^50^ and the international Guideline on Good Clinical Practice. The study also meets the General Data Protection Regulation (GDPR) criteria that came into effect on 25 May 2019.

### Adipocyte measurements with digital pathology

After sample selection, intact adipose tissue was assessed in haematoxylin and eosin (H&E) slides from 108 cases and 168 controls. In each case–control set, a case was matched with at least 1 control.

Breast adipose tissue was characterised by digital pathology using HALO^®^ image analysis software (v2.2, Indica Labs, Corrales, NM, USA). For each patient, one representative H&E slide had previously been selected for histopathological assessment and immunohistochemistry (IHC) analysis of DCIS. For the measurement of adipocyte size, areas of intact adipocytes at a distance of ≥500 µm from a DCIS duct were annotated by two experienced pathologists (M.M.A. and J.S.), blinded for case or control status. DCIS patients without intact breast adipocytes (adipocytes with intact membranes) were excluded.

Representative whole slides were scanned with a 20x objective using a Leica Aperio AT2 (Leica Microsystems, Wetzlar, Germany). To assess the relative amount of breast adipose tissue, we trained a supervised machine learning algorithm (random forest classifier) to recognise adipose, stromal and epithelial compartments from which the corresponding tissue surface and percentage were calculated.

Adipocyte diameter (µm) was digitally assessed with the HALO vacuole module v2.1 and calculated as follows: the algorithm of the HALO vacuole module calculated the centroid of the object. Subsequently, 18 diameters were passed through the centroid at different angles taken at 10-degree increments, from which the median was taken. The algorithm settings included conditions on the roundness and the regularity of the contours of the measured objects in order to discriminate between intact adipocytes and in-between areas. The same algorithm settings were used for all patients.

Measurements with a diameter under 30 µm were not considered in our analysis in order to discriminate mature adipocytes from artefacts^36^. To guarantee the measurement of true and intact adipocytes, we systematically determined adipocyte size at the 75^th^ percentile of the adipocyte population of each patient as the unique value characterising adipocyte size of that patient. Taken together, digital breast adipose characterisation resulted in percentage of adipose tissue, adipocyte diameter at the 75^th^ percentile (adipocyte diameter^75th^) and adipocyte surface area at the 75^th^ percentile (adipocyte area^75th^) for each patient.

### Histopathology and immunohistochemistry

H&E slides, histopathological data as well as ER, PR, HER2 and COX-2 IHC data were already available as previously described^13^. Antibodies are listed in Supplementary Table 1.

CD68 IHC analysis of 56 FFPE DCIS samples was performed on a BenchMark Ultra autostainer. Briefly, paraffin sections were cut at 3 µm, heated at 75 °C for 28 min and deparaffinized in the instrument with EZ prep solution (Ventana Medical Systems). Heat-induced antigen retrieval was carried out using Cell Conditioning 1 (CC1, Ventana Medical Systems) for 32 min at 95 °C. CD68 was detected using clone KP1 (Cat. No. M0814, 1/10,000 dilution, 32 min at 37 °C, Agilent / DAKO). Bound antibody was detected using the OptiView DAB Detection Kit (Ventana Medical Systems). Slides were counterstained with Haematoxylin and Bluing Reagent (Ventana Medical Systems). IHC stained slides were scanned using an Aperio AT2 Slide Scanner (Leica Microsystems, Wetzlar, Germany).

A CLS was defined as an adipocyte surrounded by a rim of CD68-positive macrophages. In order to investigate sufficient amounts of breast adipose tissue, one section of CD68 IHC data were generated on five different FFPE blocks per patient^39,45,51,52^. At least five tissue blocks were available in a subset of 56 DCIS patients (26 cases and 30 controls). Number of CLS per 10 cm^2^ of adipose tissue as a measure of adipocyte inflammation was assessed by an experienced pathologist (M.M.A.).

### Statistical analyses

Conditional logistic regression was used to estimate associations of adipocyte size, relative area of adipose tissue and number of CLS/10 cm^2^ adipose tissue with subsequent iIBC risk after primary DCIS. ORs were calculated as estimates of relative risks (RR); while 95% confidence intervals (95% CI) were Wald-based and *P*-values were likelihood ratio-based. We subsequently assessed whether adipocyte size remained an independent predictor of subsequent iIBC risk when accounting for the association of other histopathological (dominant growth pattern, grade, necrosis, calcifications, periductal fibrosis and periductal lymphocytes) and immunohistochemical features (ER, PR, HER2 and COX-2) with iIBC risk. Margin status and lesion size were excluded from the multivariable models because they were not always reliably reported in these older patient series and part of data was missing. Variables showing association with IBC risk at a *P*-value ≤ 0.1 were included in a multivariable model. Model fit was assessed by likelihood ratio tests or Akaike information criterion, in case of non-nested models. We also evaluated whether variables with a main effect in multivariable analysis modified the association of adipocyte size with subsequent iIBC risk by adding an interaction-term to our model.

BMI and menopausal status were not available. The following age categories were used to approximate menopausal status: <45 years (premenopausal), ≥45–55 years (perimenopausal) and ≥55 years (postmenopausal). Both relative area of breast adipose tissue and adipocyte size were correlated with age using these age categories as a proxy for menopausal status in all unique patients (*n* = 249). The Shapiro-Wilk test was used to assess departure from the normality assumption. Relative area of adipose tissue was compared between age categories using the non-parametric Mann-Whitney *U* test and the Kruskal-Wallis test. For comparisons of adipocyte size with age the student’s *t*-test and one-way ANOVA test was used. Correlation of the number of CLS/10 cm^2^ of adipose tissue and adipocyte size was assessed by the Spearman’s rho test.

We estimated the expected cumulative incidence of breast cancer for our population from the expected breast cancer incidence and expected all-cause mortality based on the Hakulinen method^53^ and using age-specific breast cancer incidence and mortality in the Dutch female population. All-cause mortality rates were derived from the Central Bureau of Statistics in The Netherlands while age-specific breast cancer incidence was provided by the nationwide Dutch Cancer Registry (NKR), using 5-year band for age 1-year band for calendar year.

Statistical analyses were two-sided and *P*-values < 0.05 were considered statistically significant. Analyses were performed using Stata/SE (version 13.1, StataCorp), software packages SPSS for Windows version 25.0 (IBM Corp., Armonk, NY, USA) and Graph Pad Prism 7.0 (Graph Pad Prism Inc., San Diego, CA, USA). Beeswarm plots to represent adipocyte size distribution of individual patients were performed in R (software version 4.0.3, R Development Core Team, Vienna, Austria; http://www.r-project.org) using ggplot2 and dplyr packages.

### Reporting summary

Further information on research design is available in the Nature Research Reporting Summary linked to this article.

## Supplementary information

Supplemental material.

Reporting Summary Checklist.

## Data Availability

Histopathology and immunohistochemistry data, and data on adipocyte measurements using digital pathology, which support the findings of this study, are not publicly available in order to protect patient privacy. The data will be made available upon reasonable request from the corresponding author, Prof. Jelle Wesseling, email address: j.wesseling@nki.nl. The data generated and analysed during this study are described in the following metadata record: 10.6084/m9.figshare.13580531^54^

## Code availability

Codes of Stata/SE (version 13.1. StataCorp) and R (software version 4.0.3) are available upon request. Information about the machine learning algorithms of HALO (version 2.2, Indica Labs, Corrales, NM, USA) and the Vacuole module version 2.1 is available upon request. Please contact the corresponding author.
