# Supplementary material for: Breast adipocyte size associates with ipsilateral invasive breast cancer risk after ductal carcinoma in situ
Source: NPJ Breast Cancer. 2021 Mar 22;7:31. doi: 10.1038/s41523-021-00232-w (PMC7985299; doi:10.1038/s41523-021-00232-w)
Supplement: Supplementary file 1 — Supplemental material. [file 41523_2021_232_MOESM1_ESM.pdf]

Supplementary tables and figures

Supplementary table 1 Source and dilutions of primary antibodies

Antigen	Clone	Dilution	Manufacturer
ER	SP1	Ready-to-use	Ventana
PR	1E2	Ready-to-use	Ventana
Her2	4B5	Ready-to-use	Ventana
COX-2	CX294	1/100	DAKO
CD68	KP1	1/10000	DAKO

Supplementary Table 2 Univariate results of number of CLS per 10 cm^2 with subsequent iIBC

Characteristics	Range	DCIS cases (n=108) (%)	DCIS controls (n=168) (%)	OR (95% CI)[1]	*P*[2]
CLS/10 cm^{2*}	0-211			1.24 (0.80-1.93) per 10 CLS	.16
CLS/10 cm^{2*}					.220
< 5 CLS/10 cm^2	0-5	16 (14.8)	20 (11.9)	1.00 (reference)	
≥ 5 CLS/10 cm^2	5-211	10 (9.3)	10 (6.0)	1.20 (0.43-3.35)	
N/A		82 (75.9)	138 (82.1)		

[1]: DCIS cases and DCIS controls were compared by univariate conditional logistic regression.

[2]: p-values are likelihood ratio based.

*: CLS were assessable in a 26 matched case-control sets consisting of 26 cases and 30 controls.

N/A: an insufficient number of paraffin blocks resulted in many incomplete case-control sets available for conditional logistic regression. N/As were not included in the analysis.

Supplementary Table 3 Multivariate results of adipocyte characteristics with subsequent iIBC

Characteristics	DCIS cases (n=108) (%)	DCIS controls (n=168) (%)	OR (95% CI)[1]	*P*[2]
Adipocyte area^{75th}/periductal fibrosis/ COX-2				<.001
Adipocyte area^{75th}				
Quartile 1-3	74 (68.5)	133 (79.2)	1.00 (reference)	
Quartile 4	34 (31.5)	35 (20.8)	2.20 (1.15-4.21)	
COX-2				
Low	10 (9.3)	36 (21.4)	1.00 (reference)	
High	96 (88.9)	128 (76.2)	4.29 (1.77-10.4)	
N/A	2 (1.9)	4 (2.4)		
Periductal fibrosis				
Absent	70 (64.8)	125 (74.4)	1.00 (reference)	
Present	38 (35.2)	43 (25.6)	1.54 (0.86-2.76)	
Adipocyte area^{75th}/Her2/ COX-2				<.001
Adipocyte area^{75th}				
Quartile 1-3	74 (68.5)	133 (79.2)	1.00 (reference)	
Quartile 4	34 (31.5)	35 (20.8)	2.48 (1.27-4.83)	
COX-2				
Low	10 (9.3)	36 (21.4)	1.00 (reference)	
High	96 (88.9)	128 (76.2)	4.10 (1.70-9.87)	
N/A	2 (1.9)	4 (2.4)		
Her2				
Negative	69 (63.9)	123 (73.2)	1.00 (reference)	
Positive	37 (34.3)	43 (25.6)	1.37 (0.75-2.50)	
N/A	2 (1.9)	2 (1.2)		

[1]: DCIS cases and DCIS controls were compared by multivariate conditional logistic regression.

[2]: p-values are likelihood ratio based.

Adipocyte area^{75th}, COX-2, periductal fibrosis and Her2 were included in multivariate analyses.

N/A: Not assessable; N/As were not included in the analysis.

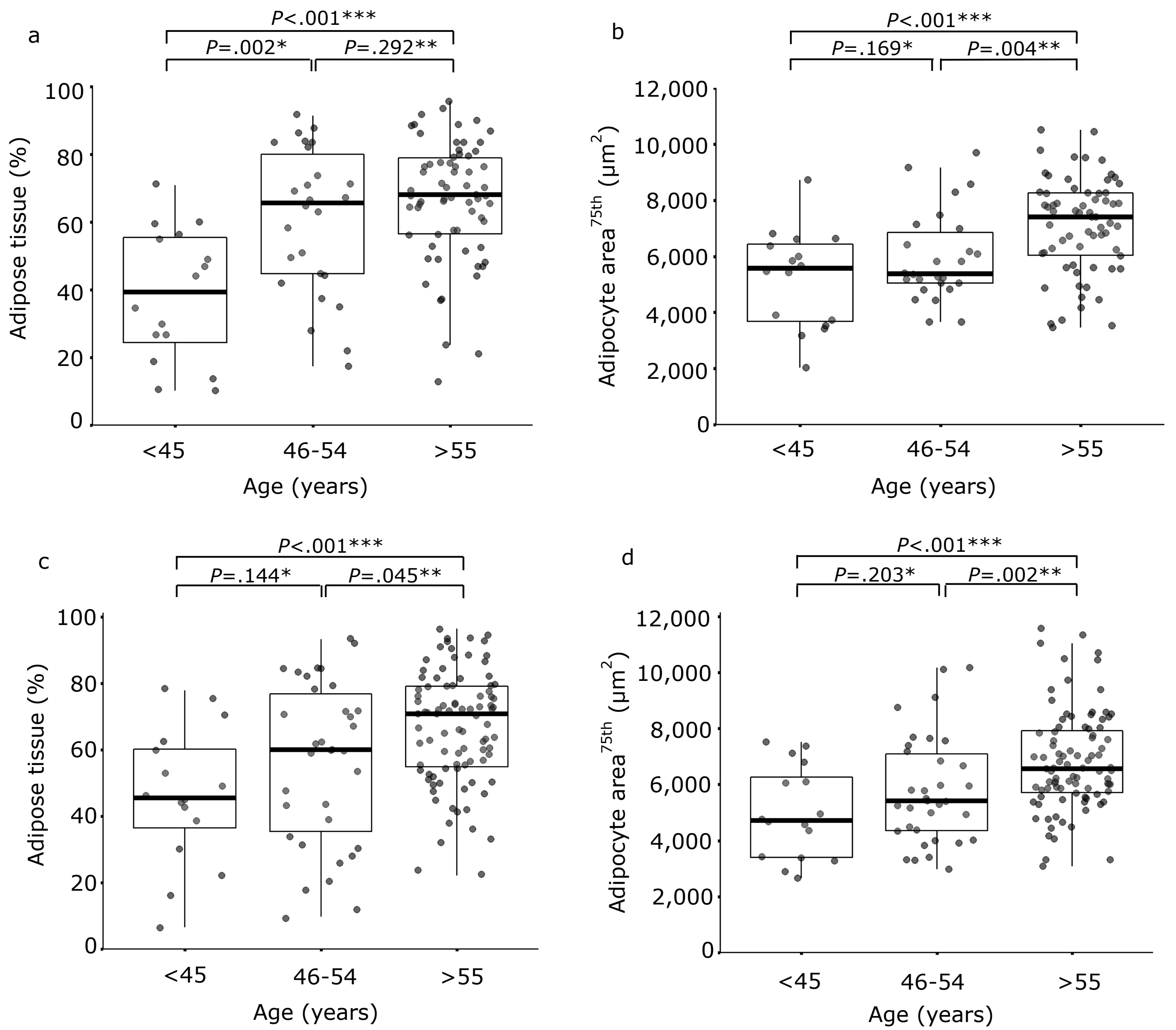

Supplementary figure 1 Older DCIS cases and controls have a higher percentage of breast adipose tissue and larger adipocyte size

a. A higher percentage of mammary adipose tissue is significantly associated with older age in DCIS cases (n=108) that developed subsequent iIBC.

*) P=.002, **) P= .292, ***) P<.001 (Mann-Whitney U Test), overall P<.001 (Kruskal-Wallis test).

b. Larger mammary adipocyte size is significantly associated with older age in cases (n=108).

*) P=.169, **) P<.004, ***) P<.001 (Student's T-test), overall P<.001 (one-way ANOVA).

c. A higher percentage of mammary adipose tissue is significantly associated with older age in DCIS controls (n=168).

*) *P*=.144, **) *P*= .045, ***) *P*<.001 (Mann-Whitney *U* Test), overall *P*<.001 (Kruskal-Wallis test).

d. Larger mammary adipocyte size is also significantly associated with older age in controls (n=168).

*) *P*=.203, **) *P*<.002, ***) *P*<.001 (Mann-Whitney *U* Test), overall *P*<.001 (Kruskal-Wallis test).

The central line in boxes represent the median value, boundaries of boxes represent the interquartile range (IQR), and ends of whiskers represent values at 1.5 x IQR.

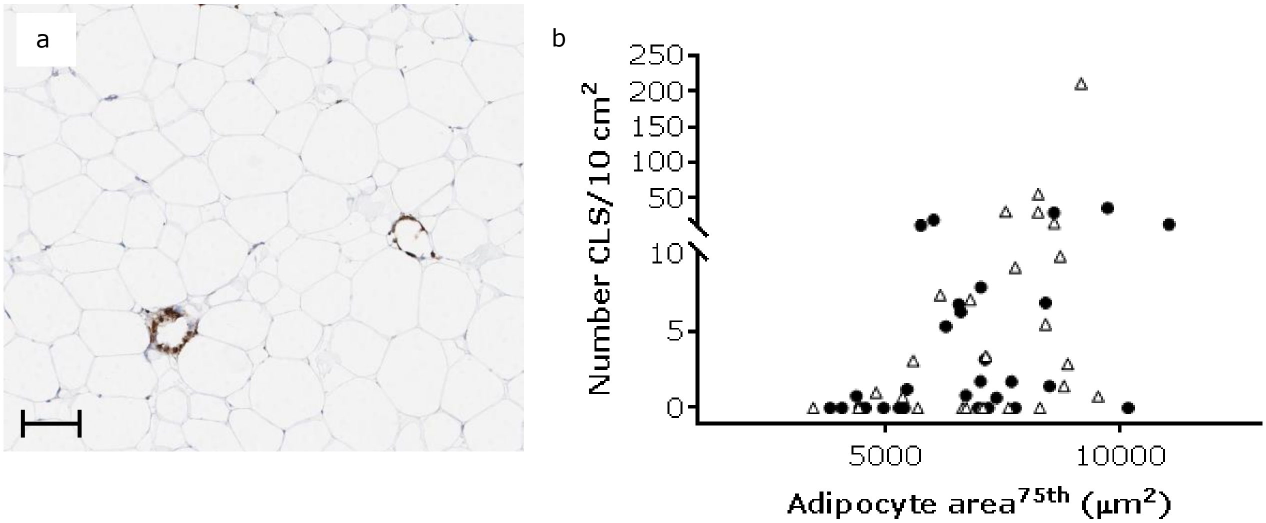

Supplementary Figure 2 Number of CLS per 10 cm^2 adipose tissue in relation to mammary adipocyte $area^{75th}$

a. High magnification of CD68 staining on mammary adipocytes with two crown-like structures. The scale bar represents 100 μm.

b. Spearman correlation coefficient was used to evaluate the relation between CLS and mammary adipocyte $area^{75th}$ in DCIS cases (triangles) and controls (black dots). The number of CLS is associated with larger adipocyte $area^{75th}$ (ρ=.47, 95% CI 0.23-0.66, *P* <.001).
