# Supplementary material for: Breast adipocyte size associates with ipsilateral invasive breast cancer risk after ductal carcinoma in situ
Source: NPJ Breast Cancer. 2021 Mar 22;7:31. doi: 10.1038/s41523-021-00232-w (PMC7985299; doi:10.1038/s41523-021-00232-w)
Supplement: Supplementary file 2 — Reporting Summary Checklist. [file 41523_2021_232_MOESM2_ESM.pdf]

nature research

Corresponding author(s): Prof. Jelle Wesseling, MD PhD

Last updated by author(s): Nov 17, 2020

Reporting Summary

Nature Research wishes to improve the reproducibility of the work that we publish. This form provides structure for consistency and transparency in reporting. For further information on Nature Research policies, see our Editorial Policies and the Editorial Policy Checklist.

Statistics

For all statistical analyses, confirm that the following items are present in the figure legend, table legend, main text, or Methods section.

n/a	Confirmed	
☐	☒	The exact sample size (n) for each experimental group/condition, given as a discrete number and unit of measurement
☐	☒	A statement on whether measurements were taken from distinct samples or whether the same sample was measured repeatedly
☐	☒	The statistical test(s) used AND whether they are one- or two-sided<br>*Only common tests should be described solely by name; describe more complex techniques in the Methods section.*
☐	☒	A description of all covariates tested
☐	☒	A description of any assumptions or corrections, such as tests of normality and adjustment for multiple comparisons
☐	☒	A full description of the statistical parameters including central tendency (e.g. means) or other basic estimates (e.g. regression coefficient) AND variation (e.g. standard deviation) or associated estimates of uncertainty (e.g. confidence intervals)
☐	☒	For null hypothesis testing, the test statistic (e.g. F, t, r) with confidence intervals, effect sizes, degrees of freedom and P value noted<br>*Give P values as exact values whenever suitable.*
☒	☐	For Bayesian analysis, information on the choice of priors and Markov chain Monte Carlo settings
☒	☐	For hierarchical and complex designs, identification of the appropriate level for tests and full reporting of outcomes
☐	☒	Estimates of effect sizes (e.g. Cohen's d, Pearson's r), indicating how they were calculated

Our web collection on statistics for biologists contains articles on many of the points above.

Software and code

Policy information about availability of computer code

Data collection	The database was assembled by M.M.A, M.S. and L.L.V.. Data of the population based cohort was retrieved from the Netherlands Cancer Registry (NCR) and the Dutch National Pathology Automated Archive (PALGA) after approval of the NCR, PALGA and institutional review boards. The study also meets the General Data Protection Regulation (GDPR) criteria. No custom algorithm or software was used.
Data analysis	Analyses were done using R (version 4.0.3), Stata/SE (version 13.1) and SPSS for Windows version 25.0

For manuscripts utilizing custom algorithms or software that are central to the research but not yet described in published literature, software must be made available to editors and reviewers. We strongly encourage code deposition in a community repository (e.g. GitHub). See the Nature Research guidelines for submitting code & software for further information.

Data

Policy information about availability of data

All manuscripts must include a data availability statement. This statement should provide the following information, where applicable:

- Accession codes, unique identifiers, or web links for publicly available datasets
- A list of figures that have associated raw data
- A description of any restrictions on data availability

Histopathology and immunohistochemistry data, and data on adipocyte measurements using digital pathology that support the findings of this study, are not publicly available in order to protect patient privacy. The data will be made available upon reasonable request from the corresponding author, Prof. Jelle Wesseling, email address: j.wesseling@nki.nl. The data generated and analysed during this study are described in the following metadata record: https://doi.org/10.6084/m9.figshare.13580531.54

Field-specific reporting

Please select the one below that is the best fit for your research. If you are not sure, read the appropriate sections before making your selection.

☒ Life sciences ☐ Behavioural & social sciences ☐ Ecological, evolutionary & environmental sciences

For a reference copy of the document with all sections, see nature.com/documents/nr-reporting-summary-flat.pdf

Life sciences study design

All studies must disclose on these points even when the disclosure is negative.

Sample size	We analyzed DCIS lesions from 276 ductal carcinoma in situ (DCIS) patients that underwent breast conserving surgery without radiotherapy from different pathology laboratories in The Netherlands, and for which formalin-fixed paraffin-embedded samples were available.
Data exclusions	See criteria described above
Replication	NA
Randomization	NA
Blinding	During digital adipose tissue segmentation and adipocyte size measurements, J.S. and M.M.A. were blinded for case- or control status

Reporting for specific materials, systems and methods

We require information from authors about some types of materials, experimental systems and methods used in many studies. Here, indicate whether each material, system or method listed is relevant to your study. If you are not sure if a list item applies to your research, read the appropriate section before selecting a response.

Materials & experimental systems

n/a	Involved in the study	
☐	☒	Antibodies
☒	☐	Eukaryotic cell lines
☒	☐	Palaeontology and archaeology
☒	☐	Animals and other organisms
☐	☒	Human research participants
☐	☒	Clinical data
☒	☐	Dual use research of concern

Methods

n/a	Involved in the study	
☒	☐	ChIP-seq
☒	☐	Flow cytometry
☒	☐	MRI-based neuroimaging

Antibodies

Antibodies used	
Validation	*Describe the validation of each primary antibody for the species and application, noting any validation statements on the manufacturer's website, relevant citations, antibody profiles in online databases, or data provided in the manuscript.*

Human research participants

Policy information about studies involving human research participants

Population characteristics	Data of the population based cohort were retrieved from the Netherlands Cancer Registry (NCR) and the Dutch National Pathology Automated Archive (PALGA)
Recruitment	This is a retrospective study that considered 276 patients
Ethics oversight	Data of the population based cohort were retrieved from the Netherlands Cancer Registry (NCR) and the Dutch National Pathology Automated Archive (PALGA) after approval of the NCR and PALGA review boards. The secondary use of tissue and data in this study are covered by an opt-out regimen conform Dutch regulations, the Code of Conduct of Federa-COREON and the international Guideline on Good Clinical Practice. The study also meets the General Data Protection Regulation (GDPR).

Note that full information on the approval of the study protocol must also be provided in the manuscript.

Clinical data

Policy information about clinical studies

All manuscripts should comply with the ICMJE guidelines for publication of clinical research and a completed CONSORT checklist must be included with all submissions.

Clinical trial registration	NA
Study protocol	NA
Data collection	NA
Outcomes	NA
